# Guided Wave Based Crack Detection in the Rivet Hole Using Global Analytical with Local FEM Approach

**DOI:** 10.3390/ma9070602

**Published:** 2016-07-21

**Authors:** Md Yeasin Bhuiyan, Yanfeng Shen, Victor Giurgiutiu

**Affiliations:** 1 Department of Mechanical Engineering, University of South Carolina, Columbia, SC 29208, USA; mbhuiyan@email.sc.edu; 2 University of Michigan-Shanghai Jiao Tong University Joint Institute, Shanghai Jiao Tong University, Shanghai 200240, China; yanfeng.shen@sjtu.edu.cn

**Keywords:** structural health monitoring, guided wave, damage detection, scatter cube, non-axisymmetric guided waves, sensor design

## Abstract

In this article, ultrasonic guided wave propagation and interaction with the rivet hole cracks has been formulated using closed-form analytical solution while the local damage interaction, scattering, and mode conversion have been obtained from finite element analysis. The rivet hole cracks (damage) in the plate structure gives rise to the non-axisymmetric scattering of Lamb wave, as well as shear horizontal (SH) wave, although the incident Lamb wave source (primary source) is axisymmetric. The damage in the plate acts as a non-axisymmetric secondary source of Lamb wave and SH wave. The scattering of Lamb and SH waves are captured using wave damage interaction coefficient (WDIC). The scatter cubes of complex-valued WDIC are formed that can describe the 3D interaction (frequency, incident direction, and azimuth direction) of Lamb waves with the damage. The scatter cubes are fed into the exact analytical framework to produce the time domain signal. This analysis enables us to obtain the optimum design parameters for better detection of the cracks in a multiple-rivet-hole problem. The optimum parameters provide the guideline of the design of the sensor installation to obtain the most noticeable signals that represent the presence of cracks in the rivet hole.

## 1. Introduction

The detection of various types of defects in structures—for example, corrosion, cracks, impact, and disbands—is an important research area of structural health monitoring (SHM) and nondestructive evaluation (NDE). Development of corrosion and fatigue cracks at the rivet holes and fasteners in the aircraft structures is the most frequent problem encountered in aircraft maintenance. The cracks can grow to a critical size and jeopardize the structural integrity if they remain undetected. Ultrasonic guided wave methods can be employed instead of the laborious point-by-point inspection method for fast, accurate, and efficient detection of the crack inauguration in the riveted holes.

### 1.1. Motivation of the Research Work

Over the topical years, the detection of cracks around the rivet hole has become an important topic of the NDE reasearch field. In 2009, the probability of detection (POD) by a model-assisted approach has been demonstrated for the fatigue crack growth in wing lap joint, wing skin fastener holes, and airframe fastener holes [1]. In 2012, the use of the transfer function approach to model-assisted POD was investigated by Bode et al. [2] through the inspection of a specimen of aircraft lap joint. However, the researches emphasized the detection of fastener hole cracks mainly based on nondestructive inspection (NDI) technique. In 2015, the SHM-based POD was obtained for the fatigue crack initiation in the lug with a wing attachment which acted as a representative airplane component [3]. The excitation signals of 200–1000 kHz center frequency were used to analyze the guided waves within fundamental Lamb wave modes. In 2014, Fu-Kuo Chang group [4] used ultrasonic SHM techniques to detect the damage and showed the variation of damage index with crack size. They determined the most influencing parameters to the sensitivity of damage detection and compared the SHM and NDE techniques. A set of transmitter and receiver sensors were used around the cracked rivet hole. However, the study of the proper location of the installed sensors around the damage was not described. The motivation and importance of the present research work has been derived from these researches.

### 1.2. State of the Art

Structural health monitoring techniques are increasingly being used for the damage detection and characterization in the aerospace structures [5,6,7]. The scattering of Lamb waves from various types of damage has been analyzed by many researchers [8,9,10,11]. Norris et al. [12] studied scattering of flexural waves on thin plates and used optical theorem to obtain far field scattering from a circular hole. They showed the azimuthal variation of the scattered flexural wave amplitude. A statistical approach to optimal sensor placement for SHM has been studied by Eric et al. [13] and their approach in the active sensing methods to three different types of plates have been demostrated. Bayes risk minimization implemented through the genetic algorithm (GA) has been used to find out the optimal arrangement of the transducers [14]. The statistical model parameters were determined experimentally to avoid the difficulty in modeling the mechanical behavoiur of the individual transducers. However, artificial surface damages were generated at different locations of the plate to implement their statistical approach. Paul Fromme et al. [15] used analytical-finite difference method (FDM) simulation to obtain the scattered field around the cracks at rivet holes, presented results at two different center frequencies for excitation, and compared these with experimental results. Lamb waves propagating in an infinite plate containing a circular hole, with or without edge cracks, were investigated by Chang and Mal [16] using a hybrid method called the global local finite element method (FEM). However, the research work was limited to the symmetric Lamb modes and the incident Lamb wave mode was perpendicular to the crack. Recent researches have put emphasis on the simulated results using fast and efficient numerical techniques to understand the Lamb wave behavior prior to implementation of the results in the physical structures [17]. Numerical methods are becoming a popular tool for understanding the complex Lamb wave interaction with complicated boundary conditions [18,19]. The scatter field of a single rivet hole cracks with a single directed (incident) Lamb wave has been described by introducing the wave damage interaction coefficient (WDIC), and the non-reflective boundary (NRB) was implemented to simulate the infinite plate in a successful manner [20].

In the present research, the optimal placement of the transducer—as well as the excitation frequency for the actuator in an active sensing method of SHM—has been obtained based on the wave scattering phenomenon. The wave scattering phenomenon is obtained using the FEM while the wave propagation is modeled using exact analytical formulations.

### 1.3. Scope of the Article

In this article, the problem of Lamb wave scattering is explored more in the perspective of analyzing multiple-rivet-hole lap joint cracks. The detection of cracks in a multiple-rivet-hole lap joint is considered by analytical-FEM simulation, which is an effective tool for the analysis in contrast to costly experimental investigation. Harmonic analysis is performed to the small-size 3-D model of the local damage using FEM. Both symmetric and anti-symmetric Lamb wave modes incident from multiple driections on the damage are analyzed and scattered coefficients are calculated around the damage corresponding to each incident direction. Wave-damage interaction coefficients (WDICs) are used to describe the scattering behavior of the damage and “scatter cubes” are formed for both Lamb wave modes. The transfer function, based on an analytical model accompanied by “scatter cube”, is used to find the optimum center frequency of the toneburst signal from the actuator and the most damage-sensitive location in the structure where the sensor can be installed. The most sensitive signal that contains the prominent damage signature is obtained analytically. A simplified case of the real problem has been demonstrated using the simulated signals obtained through the combined analytical and FEM method.

## 2. Description of the SHM of Multiple-Rivet-Hole Lap Joint

**Figure 1:**
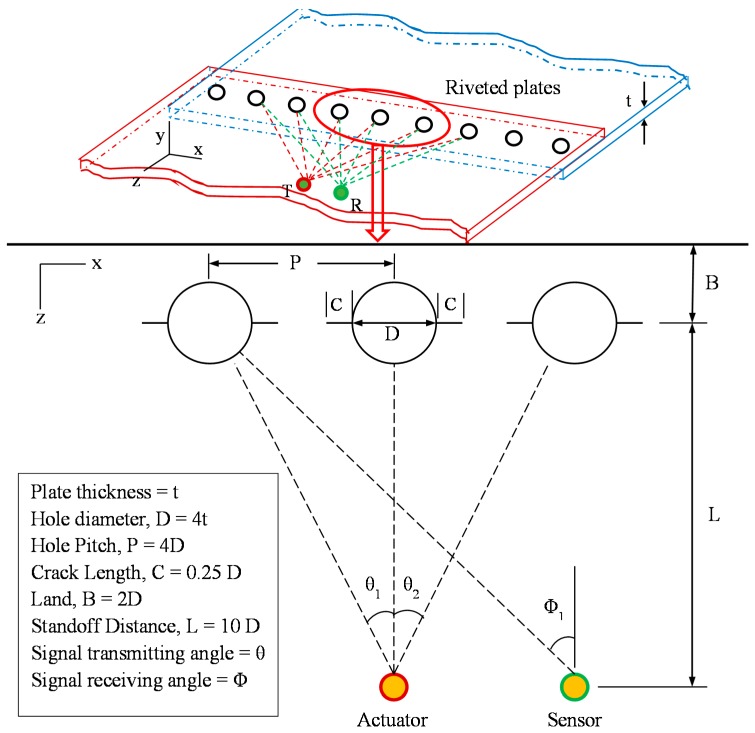
Illustration of the multiple-rivet-hole lap joint.

A multiple-rivet-hole lap joint with active transmitting-sensing sensors of an SHM system is illustrated in Figure 1. When electrical voltage is supplied to the piezoelectric transmitter (actuator), it generates mechanical excitation in the structure and produces Lamb waves that propagate in the structure. The Lamb waves interact with the damages, which act as the secondary sources of guided waves. The scattered guided waves propagate in the structures and are received by the piezoelectric sensor. The actuator dispatches Lamb waves to each of the rivet holes at a certain angle that can be calculated from the standoff distance (L) and pitch (P) of each rivet hole (Figure 1). The scattering phenomenon depends on the direction (θ) of incident Lamb waves as well as the azimuth direction (Φ) around the damage (the azimuth direction Φ corresponds to the sensor placement direction).

The secondary source (cracked rivet hole) is asymmetric for a certain angle of incident Lamb waves, hence, it acts as a non-axisymmetric secondary source of scattered waves. The scattered waves contain scattered Lamb waves and shear horizontal (SH) waves. Understanding the non-axisymmetric scattered waves around the damage provides the ability to better detect the emanating cracks in the rivet holes. The actuator and sensor installment, as well as the excitation frequency, depend on the non-axisymmetric behavior of the scattered guided waves. The design of proper transducer installation and selection of the center frequency of excitation enable capturing the better damage signature originating from the cracks of the rivet holes.

Since no closed form solution exists for the non-axisymmetric scattered waves, a combined analytical and finite element modeling has been introduced in the present article. Exact closed form analytical formulation has been used for the propagation of Lamb waves from the actuator up to the damage in the structure. The interaction of the Lamb waves with the local damage is modeled using finite element analysis and the non-axisymmetric behaviors of the scattered waves is captured through the WDICs and scatter cubes.

## 3. Analytical Modeling of Lamb Wave Propagation up to Local Damage

Lamb waves are generated from the actuator which is an axisymmetric primary source of Lamb waves. Hence, for the incident Lamb wave modeling the axisymmetric condition applies.

### 3.1. Lamb Wave Generation from the Actuator

Lamb waves are generated in the plate by a circular actuator and propagate toward the local damage. For outward propagating waves from the actuator, the radial displacement $u_{r}(r)$ on the top surface of the plate at any distance *r* from the actuator is given by Giurgiutiu [21], i.e.,

(1) $${u_{r}(r)|}_{z=d}=-\frac{\pi i}{2\mu }\sum_{j=0}^{J_{S}}\frac{\tilde{\tau }(\xi_{j}^{S})N_{S}(\xi_{j}^{S})}{{D^{\prime }}_{S}(\xi_{j}^{S})}H_{1}^{\left(1\right)}(\xi_{j}^{S}r)e^{-i\omega t}-\frac{\pi i}{2\mu }\sum_{j=0}^{J_{A}}\frac{\tilde{\tau }(\xi_{j}^{A})N_{A}(\xi_{j}^{A})}{{D^{\prime }}_{A}(\xi_{j}^{A})}H_{1}^{\left(1\right)}(\xi_{j}^{A}r)e^{-i\omega t}$$

where μ=G, is the shear modulus of the plate, *a* is the radius of actuator, and the components $N_{S},N_{A},D_{S},D_{A}$ can be defined as

(2) $$\begin{array}{l} N_{S}\left(\xi \right)=\xi \eta_{S}\left(\xi^{2}+\eta_{S}^{2}\right)\cos\eta_{P}d\cos\eta_{S}d;\;N_{A}\left(\xi \right)=-\xi \eta_{S}\left(\xi^{2}+\eta_{S}^{2}\right)\sin\eta_{P}d\sin\eta_{S}d\\ D_{S}={\left(\xi^{2}-\eta_{S}^{2}\right)}^{2}\cos\eta_{P}d\sin\eta_{S}d+4\xi^{2}\eta_{P}\eta_{S}\sin\eta_{P}d\cos\eta_{S}d\\ D_{A}={\left(\xi^{2}-\eta_{S}^{2}\right)}^{2}\sin\eta_{P}d\cos\eta_{S}d+4\xi^{2}\eta_{P}\eta_{S}\cos\eta_{P}d\sin\eta_{S}d \end{array}$$

where 2*d* is the thickness of the plate; $\eta_{P}$, $\eta_{S}$ can be defined as

(3) $$\eta_{P}^{2}=\frac{\omega^{2}}{c_{P}^{2}}-\xi^{2};\;\eta_{S}^{2}=\frac{\omega^{2}}{c_{S}^{2}}-\xi^{2}$$

The wavespeed $c_{P}$ and $c_{S}$ depend on the plate material properties (Lame constants λ, μ and density ρ)

(4) $$c_{P}=\sqrt{\frac{\lambda +2\mu }{\rho }};\;c_{S}=\sqrt{\frac{\mu }{\rho }}$$

Considering the actuator is ideally bonded to the plate of thickness = 2*d*, and $\tau_{a}$ be the shear stress between the plate and the transducer, the $J_{1}$ Hankel transform of the radial shear stress can be written as

(5) $$\tilde{\tau }{\left(\xi \right)}_{J_{1}}=\tau_{a}a^{2}J_{1}(\xi a)$$

where, $J_{1}$ is the first order Bessel function.

Substituting Equation (5) into Equation (1) yields

(6) $$\begin{array}{cc} {u_{r}(r)|}_{z=d}= & -\pi i\frac{a^{2}\tau_{a}}{2\mu }\sum_{\xi^{S}}\frac{J_{1}(\xi^{S}a)N_{S}(\xi^{S})}{{D^{\prime }}_{S}(\xi_{j}^{S})}H_{1}^{\left(1\right)}(\xi^{S}r)e^{-i\omega t}\\ & -\pi i\frac{a^{2}\tau_{a}}{2\mu }\sum_{\xi^{A}}\frac{J_{1}(\xi^{A}a)N_{A}(\xi^{A})}{{D^{\prime }}_{A}(\xi^{A})}H_{1}^{\left(1\right)}(\xi^{A}r)e^{-i\omega t} \end{array}$$

The first kind Hankel function of order one $H_{1}^{\left(1\right)}$ represents outgoing propagating waves. The wavenumber ξ depends on the frequency and is the roots of the Rayleigh–Lamb equation [22], i.e.,

(7) $$\frac{\tan\eta_{S}d}{\tan\eta_{P}d}={\left[\frac{-4\eta_{P}\eta_{S}\xi^{2}}{{\left(\xi^{2}-\eta_{S}^{2}\right)}^{2}}\right]}^{\pm 1}$$

where, +1 and −1 is for symmetric and antisymmetric Lamb wave modes, respectively.

### 3.2. Actuator Transfer Function

The piezoelectric wafer active sensor (PWAS) acts as an actuator which is supplied with a voltage input. The PWAS transfer function $g_{PWAS}\left(\omega \right)$ relates the applied voltage ${\tilde{V}}_{T}(\omega )$ to shear stress $\tau_{a}$ and defined as

(8) $$F_{a}=a\tau_{a}=g_{PWAS}\left(\omega \right){\tilde{V}}_{T}(\omega )$$

where $F_{a}$ is the reaction force per unit length from the structure due to the expansion of PWAS mounted on the surface of the structure, *a* is the radius of actuator, ${\tilde{V}}_{T}(\omega )$ can be obtained by the Fourier transform of the time–domain excitation signal $V_{T}(t)$. The transfer function $g_{PWAS}\left(\omega \right)$ of the actuator can be derived as in Equation (9). The detail derivation can be found in reference [23].

(9) $$g_{PWAS}\left(\omega \right)=\frac{d_{31}}{s_{11}^{E}}\frac{r\left(\omega \right)}{1-r\left(\omega \right)}$$

where $r\left(\omega \right)=k_{str}(\omega )/k_{PWAS}$ is the stiffness ratio between host structure and actuator, $d_{31}$ is piezoelectric strain coefficient, $s_{11}^{E}$ is the mechanical compliance of the actuator material measured at zero electric field (E = 0) [23].

The WDIC obtained from the FEM analysis depends on the material properties of host structure while the final signal received depends on both the transducer material properties and host structure properties.

### 3.3. Structure Transfer Function

The roots of the Rayleigh–Lamb Equation (7) provide numerous symmetric and antisymmetric wavenumbers $\xi^{S}$, $\xi^{A}$ for a certain excitation frequency *w*. These wavenumbers are used in the summation process in Equation (6). The structural transfer function can be calculated by substitution of Equation (9) into Equation (6) and dividing by $e^{-i\omega t}$. For convenience, the symmetric (*S*) and antisymmetric (*A*) transfer function may be separated as

(10) $$G^{S}\left(\omega ,r\right)=-\pi i\frac{ag_{PWAS}\left(\omega \right)}{2\mu }\sum_{\xi^{S}}\frac{J_{1}(\xi^{S}a)N_{S}(\xi^{S})}{{D^{\prime }}_{S}(\xi^{S})}H_{1}^{\left(1\right)}(\xi^{S}r)$$

(11) $$G^{A}\left(\omega ,r\right)=-\pi i\frac{ag_{PWAS}\left(\omega \right)}{2\mu }\sum_{\xi^{A}}\frac{J_{1}(\xi^{A}a)N_{S}(\xi^{A})}{{D^{\prime }}_{S}(\xi^{A})}H_{1}^{\left(1\right)}(\xi^{A}r)$$

### 3.4. Direct Incident Signal

The structure transfer function can be multiplied by the frequency–domain excitation signal ${\tilde{V}}_{T}(\omega )$ to obtain the direct incident waves at the sensing location, i.e.,

(12) $$u_{IN}\left(\omega ,R_{IN}\right)={\tilde{V}}_{T}(\omega )\left[G^{S}\left(\omega ,R_{IN}\right)+G^{A}\left(\omega ,R_{IN}\right)\right]$$

where the distance $R_{IN}$ from actuator up to sensing location is used.

Similarly, the structure transfer function can be multiplied by ${\tilde{V}}_{T}(\omega )$ up to the damage location to obtain the interrogating waves arriving at the damage, i.e.,

(13) $$u_{D}\left(\omega ,R_{D}\right)={\tilde{V}}_{T}(\omega )\left[G^{S}\left(\omega ,R_{D}\right)+G^{A}\left(\omega ,R_{D}\right)\right]$$

where the distance $R_{D}$ from actuator up to the damage location is used.

It can be noted that the Lamb modes propagate independently, and the direct incident wave field is the superposition of symmetric and antisymmetric wave modes.

## 4. Effect of Local Damage through the Insertion of Scatter Cube of WDIC

### 4.1. Concept of WDIC

**Figure 2:**
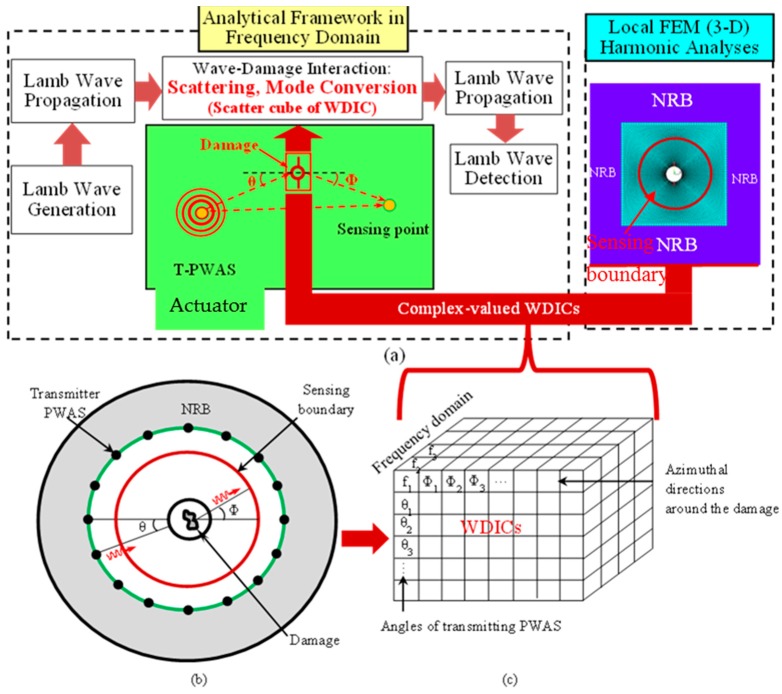
(**a**) Framework of the combined analytical and finite element approach (CAFA); (**b**) Representative model of the damage for FE analysis; (**c**) “Scatter cube” of wave damage interaction coefficient (WDIC).

The displacement field of the incident wave ($u_{IN}$) may be obtained from the center point of the pristine model. The incident displacement field coming towards the center of the damage and the scattered displacement field recorded at the sensing boundary (Figure 2a) follows a certain relation [24], i.e.,

(14) $$u_{IN}^{A}e^{-i\varphi_{IN}^{A}}C_{AB}\left(\omega ,\theta ,\Phi \right)e^{-i\varphi_{AB}\left(\omega ,\theta ,\Phi \right)}H_{m}^{\left(1\right)}\left(\xi^{B}r\right)=u_{SC}^{B}\left(\omega ,\theta ,\Phi \right)e^{-i\varphi_{SC}^{B}\left(\omega ,\theta ,\Phi \right)}$$

where $u_{IN}^{A}e^{-i\varphi_{IN}^{A}}$ represents any incident mode *A* at the center of the damage; $C_{AB}\left(\omega ,\theta ,\Phi \right)e^{-i\varphi_{AB}\left(\omega ,\theta ,\Phi \right)}$ represents the amplitude $C_{AB}\left(\omega ,\theta ,\Phi \right)$ and phase $e^{-i\varphi_{AB}\left(\omega ,\theta ,\Phi \right)}$ of WDIC; r is the radius of sensing boundary; $H_{m}^{\left(1\right)}\left(\xi^{B}r\right)$ is the Hankel function of 2-D scattered wave field (B) propagates in outward direction with *m* = 1. $C_{AB}$ stands for the incident *A* wave mode to the scattered *B* wave mode that depends on the direction of incident Lamb wave, azimuthal direction of the damage and the circular frequency. On the right side $u_{SC}^{B}\left(\omega ,\theta ,\Phi \right)e^{-i\varphi_{SC}^{B}\left(\omega ,\theta ,\Phi \right)}$ represents the scattered displacement field along the sensing boundary of radius r.

Upon rearrangement Equation (14) yields

(15) $$C_{AB}\left(\omega ,\theta ,\Phi \right)e^{-i\varphi_{AB}\left(\omega ,\theta ,\Phi \right)}=\frac{u_{SC}^{B}\left(\omega ,\theta ,\Phi \right)}{u_{IN}^{A}}\frac{1}{H_{m}^{\left(1\right)}\left(\xi^{B}r\right)}e^{-i\Delta \varphi_{AB}\left(\omega ,\theta ,\Phi \right)}$$

where, $\Delta \varphi_{AB}\left(\omega ,\theta ,\Phi \right)=\varphi_{SC}^{B}\left(\omega ,\theta ,\Phi \right)-\varphi_{IN}^{A}$. From Equation (15) the amplitude and phase may be separated as

(16) $$C_{AB}\left(\omega ,\theta ,\Phi \right)=\left|\frac{u_{sc}^{B}\left(\omega ,\theta ,\Phi \right)}{u_{IN}^{A}}\frac{1}{H_{m}^{\left(1\right)}\left(\xi^{B}r\right)}\right|$$

(17) $$\varphi_{AB}\left(\omega ,\theta ,\Phi \right)=\Delta \varphi_{AB}\left(\omega ,\theta ,\Phi \right)-\left[∠\frac{1}{H_{m}^{\left(1\right)}\left(\xi^{B}r\right)}-∠\frac{1}{H_{m}^{\left(1\right)}\left(0^{+}\right)}\right]$$

For instance, when incident symmetric Lamb wave mode (*S*0 mode) causes scattered *S*0 mode then both *A* and *B* corresponds to the parameters of *S*0 mode. When incident *S*0 mode causes scattered symmetric *SH* mode then *A* corresponds to the parameters of *S*0 mode but *B* corresponds to the *SH* wave mode.

WDIC depends on the thickness of the plate. The thickness of the upper and lower plate is considered to be the same for the present analysis. Hence, the result of the top plate will be the same for the bottom plate. That means in order to monitor the damage in the bottom plate, a similar arrangement of the transducer is needed in the top plate. In case of different thick plates, the two plates should be considered individually. Each plate should be analyzed using the proposed method to obtain the optimal configuration of the transducers for individual plates. However, there may occur some wave leakages through the bonded rivet, thus, the thickness of that bonded plate section will come into play and may modify the WDIC profile which can be addressed as well. In this present analysis, the bonded wave leakage is not considered.

### 4.2. Separation of the Modes

The displacement wave fields of the local damage model can be solved using finite element method (the details will be discussed in Section 5). The scattered wave displacements at the top and bottom surface sensing nodes in both radial ($u_{r}^{T}$ and $u_{r}^{B}$) and tangential ($u_{\theta }^{T}$ and $u_{\theta }^{B}$) directions can be used to selectively separate each wave mode, as follows:

(18) $$u_{SC}^{S0}=\frac{u_{r}^{T}+u_{r}^{B}}{2};\;u_{SC}^{A0}=\frac{u_{r}^{T}-u_{r}^{B}}{2};\;u_{SC}^{SH_{S0}}=\frac{u_{\theta }^{T}+u_{\theta }^{B}}{2};\;u_{SC}^{SH_{A0}}=\frac{u_{\theta }^{T}-u_{\theta }^{B}}{2}$$

It should be clearly noted that in this study we focused on the fundamental Lamb wave modes (*S*0 and *A*0) and fundamental shear horizontal mode (*SH*0). The frequency range of our analysis is below the cut-off frequencies of the higher Lamb and *SH* wave modes. We denote fundamental $SH_{S0}$ mode as SH mode. The extension of our approach to higher modes will be done in a future study.

### 4.3. Inserting of WDICs of Scattered Modes

The scattered displacement fields provide the scattered coefficients for the scattered wave modes ($C_{SS},C_{AS},C_{SA},C_{AA},C_{SSH},C_{ASH}$) following Equation (15). Each scattered mode forms a scatter cube considering the multiple incident directions of Lamb wave. Scattered wave source at the damage location is obtained by modifying incident waves at the damage with WDICs

(19) $$u_{N}^{S}=C_{SS}\left(\omega ,\theta ,\Phi \right)e^{-i\varphi_{SS}\left(\omega ,\theta ,\Phi \right)}u_{D}^{S}+C_{AS}\left(\omega ,\theta ,\Phi \right)e^{-i\varphi_{AS}\left(\omega ,\theta ,\Phi \right)}u_{D}^{A}$$

(20) $$u_{N}^{A}=C_{SA}\left(\omega ,\theta ,\Phi \right)e^{-i\varphi_{SA}\left(\omega ,\theta ,\Phi \right)}u_{D}^{S}+C_{AA}\left(\omega ,\theta ,\Phi \right)e^{-i\varphi_{AA}\left(\omega ,\theta ,\Phi \right)}u_{D}^{A}$$

(21) $$u_{N}^{SH}=C_{SSH}\left(\omega ,\theta ,\Phi \right)e^{-i\varphi_{SSH}\left(\omega ,\theta ,\Phi \right)}u_{D}^{S}+C_{ASH}\left(\omega ,\theta ,\Phi \right)e^{-i\varphi_{ASH}\left(\omega ,\theta ,\Phi \right)}u_{D}^{A}$$

where $u_{N}^{S}$, $u_{N}^{A}$, and $u_{N}^{SH}$ represent the damage scattered *S*0, *A*0, and *SH*0 wave source respectively. The new wave source (damage) irradiates the scattered waves that propagate to the sensing location. The 2-D Lamb wave irradiating from a point source accepts the following solution in the cylindrical coordinate system with reference to the new wave source location [21,25].

(22) $$u_{r}=\sum_{n=1}^{\infty }a_{n}^{LW}\left(z\right)H_{1}^{\left(1\right)}\left(\xi_{n}^{LW}r\right)e^{-i\omega t}$$

(23) $$u_{\theta }=\sum_{n=1}^{\infty }b_{n}^{SH}\left(z\right)H_{1}^{\left(1\right)}\left(\xi_{n}^{SH}r\right)e^{-i\omega t}$$

where $a_{n}^{LW}\left(z\right)$ and $b_{n}^{SH}\left(z\right)$ are the thickness dependent mode shapes for Lamb and *SH* waves of *n*^th^ wave mode.

### 4.4. Scattered Sensing Signals

Since the amplitude relationship between the interrogating waves and the scattered waves is enclosed in the WDICs, the transfer function from the damage up to the sensing location is simply $H_{1}^{\left(1\right)}\left(\xi R_{SC}\right)$, where $R_{SC}$ is the distance from the damage up to the sensing location. Thus, the scattered waves arriving at the sensing point can be calculated.

(24) $$u_{SC}^{S}=u_{N}^{S}H_{1}^{\left(1\right)}\left(\xi^{S}R_{SC}\right);\;u_{SC}^{A}=u_{N}^{A}H_{1}^{\left(1\right)}\left(\xi^{A}R_{SC}\right);\;u_{SC}^{SH}=u_{N}^{SH}H_{1}^{\left(1\right)}\left(\xi^{SH}R_{SC}\right)$$

Since the frequency domain excitation voltage ${\tilde{V}}_{T}(\omega )$ is used to derive the above relations, the inverse Fourier transform is used to obtain the time–domain scattered signal. The out-of-plane displacement wave field may be obtained through the multiplication of the in-plane displacements by the mode shape component ratio, and the out-of-plane velocity would be the time derivative of the out-of-plane displacement [21].

## 5. Description of the FEM Modeling

At large distances from the origin, the behavior of circular-crested Lamb waves approaches asymptotically that of straight-crested Lamb waves, but the amplitude is affected by the factor which captures the geometric spreading of the circular wave front [21]. Considering the standoff distance in the multiple-rivet-hole in Figure 1, i.e., the excitation source is far away from the damage, it is a good approximation to use straight-crested Lamb modes as incident waves in the small local damage region during the nodal load calculations of FEM. A 40 mm × 40 mm 3D FE model of a 1.6 mm thick aircraft-grade aluminum-2024-T3 plate is created using commercial ANSYS15 software (ANSYS, Inc., Canonsburg, PA, USA). A non-reflective boundary (NRB) 20 mm wide at each side of the model is used. The crack length (2*a*) to diameter (*d*) of the hole ratio of 0.5 is used.

### 5.1. Imposing the Nodal Point Load

**Figure 3:**
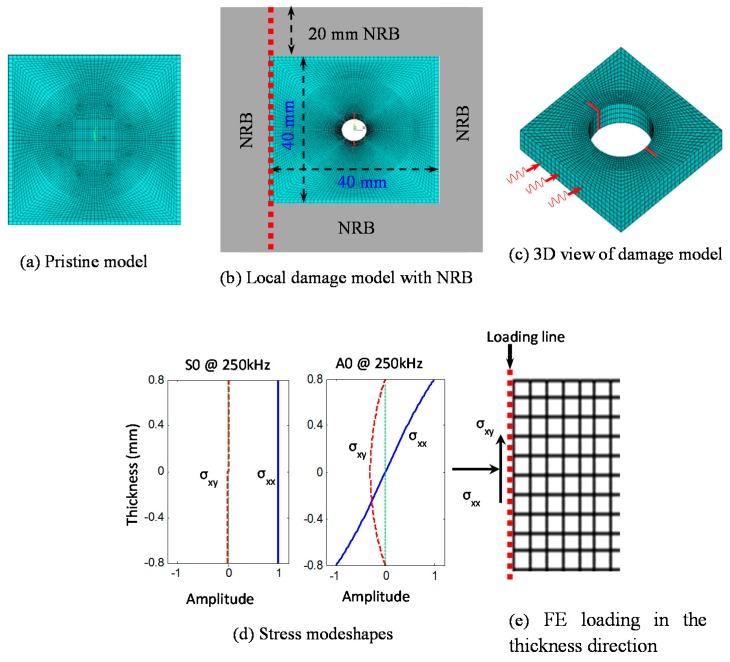
Illustration of local finite element method (FEM) modeling (**a**) Pristine model; (**b**) Local damage model with NRB; (**c**) 3D view of damage model; (**d**) Stress modeshapes; (**e**) FE Loading in the thickness direction.

The 3D view of the local FEM model is shown in Figure 3c. Incoming Lamb waves are shown as three red signal signs on one face of the model. Lamb mode excitation is imposed through nodal forces by evaluating integrals of stress mode shape components on the loading nodes. The stress mode shapes are calculated analytically [21] and converted into nodal forces through boundary integration on each element along the loading line. The element nodal force can be evaluated by using Equation (25) [26].

(25) $$F_{ix}^{e}=\int_{0}^{L_{e}}\sigma_{xx}^{e}\left(s\right)N_{i}^{e}\left(s\right)ds;\;F_{iy}^{e}=\int_{0}^{L_{e}}\sigma_{xy}^{e}\left(s\right)N_{i}^{e}\left(s\right)ds$$

where subscript and superscript e stands for element, $L_{e}$ represents the element size, $F_{ix}^{e}$ and $F_{iy}^{e}$ are nodal forces in x and y direction, i is the element node number, $N_{i}^{e}\left(s\right)$ is the shape function of selected element type. The nodal forces are updated for each calculation step, imposing Lamb mode excitation for each excitation frequency. The stress mode shapes and loading line along a single line on that face is shown in Figure 3e.

For symmetric modes:

(26) $$\begin{array}{l} \sigma_{xx}^{S}(x,y,t)=C^{S}2{\mu \xi \eta }_{S}\left[\left(\xi^{2}+\eta_{S}^{2}-2\eta_{P}^{2}\right)\cos\eta_{S}d\cos\eta_{P}y-(\xi^{2}-\eta_{S}^{2})\cos\eta_{P}d\cos\eta_{S}y\right]e^{i\left(\xi x-\omega t\right)}\\ \sigma_{xy}^{S}(x,y,t)=iC^{S}\mu \left[4\xi^{2}\eta_{P}\eta_{S}\cos\eta_{S}d\sin\eta_{P}y+{\left(\xi^{2}-\eta_{S}^{2}\right)}^{2}\cos\eta_{P}d\sin\eta_{S}y\right]e^{i\left(\xi x-\omega t\right)} \end{array}$$

For antisymmetric modes:

(27) $$\begin{array}{l} \sigma_{xx}^{A}(x,y,t)=-C^{A}2{\mu \xi \eta }_{S}\left[\left(\xi^{2}+\eta_{S}^{2}-2\eta_{P}^{2}\right)\sin\eta_{S}d\sin\eta_{P}y-(\xi^{2}-\eta_{S}^{2})\sin\eta_{P}d\sin\eta_{S}y\right]e^{i\left(\xi x-\omega t\right)}\\ \sigma_{xy}^{A}(x,y,t)=iC^{A}\mu \left[4\xi^{2}\eta_{P}\eta_{S}\sin\eta_{S}d\cos\eta_{P}y+{\left(\xi^{2}-\eta_{S}^{2}\right)}^{2}\sin\eta_{P}d\cos\eta_{S}y\right]e^{i\left(\xi x-\omega t\right)} \end{array}$$

### 5.2. Meshing, Imposing NRB, and Validation of the FE Model

Non-reflective boundary (NRB) has been employed surrounding the local FEM model (as shown in Figure 2a) in order to avoid the reflections from the edges. The adequate non-reflective boundary is implemented following our published paper in references [20,24] and is not discussed here for the sake of brevity.

Eight node structure elements (SOLID45) are used to mesh the plate, and spring-damper elements (COMBIN14) are used to construct the NRB. In the thickness direction, 0.4 mm mesh size is used, which is fair enough to capture the thickness mode shapes. Finer meshing is used in the crack region to accommodate the high stress gradient and coarser meshing is used away from the crack and outside the sensing boundary. The finite element results are validated with the results obtained for Lamb wave incident at 0° degree in reference [24].

**Figure 4:**
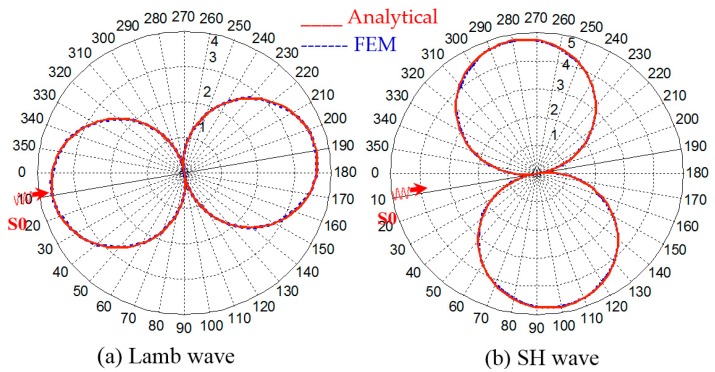
Comparison between analytical and FEM results (**a**) WDIC_LW_LW_ (**b**) WDIC_LW_SH_ in polar coordinates (pristine plate).

The analytical WDIC profiles for the Lamb wave and SH wave for a simple pristine plate has been derived by Bhuiyan et al. [27], who showed that WDIC profiles follow ideal double-circled shape. The finite element result is compared with the analytical result as shown in Figure 4. The finite element result shows very good agreement with the analytical result.

### 5.3. Modeling of the Cracks of the Rivet Hole

Cracks in the rivet hole are modeled using the discontinuity at the adjacent pair of nodes along the cracks. There are actually two sets of nodes along the crack faces, and each set is representing the nodes on each face; the nodes are discontinuous along the crack faces. Two sets of nodes are adjacent to each other unlike the modeling of notch, where there is a small gap between those sets of nodes. Like the nodes, the solid elements are also disbanded along the crack faces. The modeling of the cracks in finite element using the above approach is fair enough to model the actual cracks of rivet holes in the plate-like structure [28].

### 5.4. Selection of Frequency Domain for Harmonic Analysis

The frequency domain of harmonic analysis was selected based on dispersion curves to confine our analysis to the fundamental Lamb and SH wave modes. At very low frequencies (<40 kHz), the wavelength of the Lamb modes is very high and requires very wide NRB and, hence, requires more computation resources in FEM. It also requires a longer distance to die out the non-propagating A1 mode of Lamb wave. At higher frequencies (>900 kHz), the wavelength becomes very small and requires very fine mesh to capture the damage feature, thus requiring more time to solve the model. At higher frequencies, the higher modes (S1, A1, S2, A2, etc.) of Lamb and SH waves can appear and make the analysis more complex. Considering these reasons, the frequency domain of 40–900 kHz with a frequency step of 2 kHz was selected for the harmonic analysis.

The sensing boundary is located sufficiently far from the crack so that all non-propagating Lamb and SH scattered wave modes die out before they reach the sensing locations. Thus, the wave fields at the sensing locations around the rivet hole with cracks are the contributions of propagating Lamb and SH wave modes. Three sets of FEM simulations were carried out to find the contribution of the butterfly cracks to the WDICs: (a) Lamb wave interaction with rivet hole with butterfly cracks; (b) Lamb wave interaction with rivet hole; and (c) Lamb wave interaction with a pristine plate. In order to get the wave field at the sensing boundary due to the presence of butterfly cracks in a rivet hole, the wave field of the hole was subtracted from the combined wave field of rivet hole with butterfly cracks. The wave field of the pristine model provides the required direct incident wave fields in Equation (16). The WDICs provide the indication of getting strong or weak signals around the damage. In order to readily identify those locations, the WDICs are plotted in polar coordinate system.

## 6. Discussion of the Simulation Results

### 6.1. Formation of Scatter Cube

For each transmitting angle (θ), the WDICs are recorded at azimuthal sensing angles (Φ) around the sensing boundary over the frequency domain. The results of the harmonic analyses of the model facilitate forming a “scatter cube” of complex-valued WDICs as shown in Figure 2c. The three dimensions of the scatter cube contain the WDICs for various frequencies, angles of transmitting PWASs, and angles of sensing PWASs. These WDICs can describe complicated 3-D interaction between the interrogating waves and damage, i.e., scattering and mode conversion. Since the problem of rivet hole with cracks is symmetric with respect to the mid-plane of the thickness direction, no antisymmetric wave mode generates for symmetric Lamb wave incident, and vice versa.

### 6.2. Distortion of WDIC Profile Due to the Presence of Cracks

**Figure 5:**
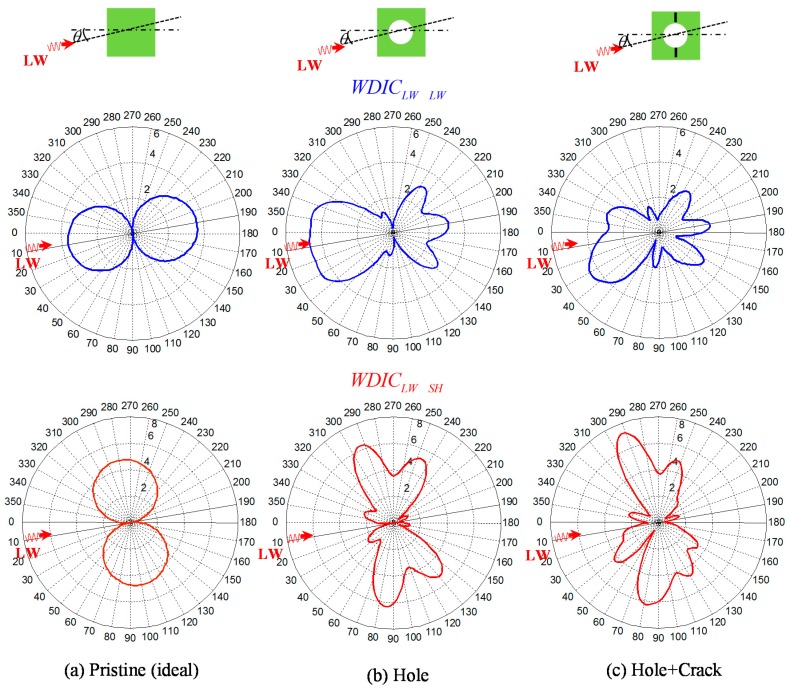
Alteration of wave damage interaction coefficient (WDIC) profiles of scattered Lamb and shear horizontal (SH) waves with different damage conditions: (**a**) Pristine; (**b**) Hole; (**c**) Hole + Crack.

**Figure 6:**
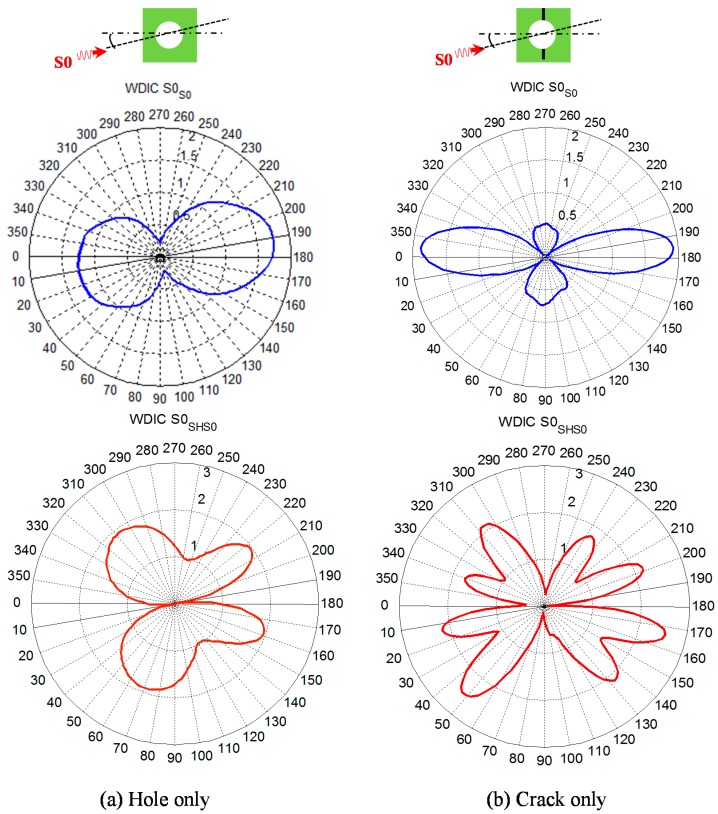
Subtracted WDIC profiles of scattered Lamb and SH waves to account the damage effect only: (**a**) Hole only; (**b**) Crack only.

The polar plot of WDIC (WDIC profile) of the scattered *S*0 Lamb mode and *SH* mode due to incident *S*0 Lamb mode at θ=9° is shown in Figure 5. An arbitrary frequency f=486 kHz was selected for illustration purposes only. When there is no damage (pristine) in the plate, there is no scattered wave field and the WDIC profile is an ideal double-circled shape. When there is a rivet hole in the plate, the presence of scattered field makes distortion of the ideal shape of WDIC profile as shown in Figure 5b and the profile is symmetric about the line of incidence since the rivet hole is symmetric about the line of incidence. When there is damage (butterfly cracks) in the rivet hole, the additional scattered waves due to damage provide additional distortion to the WDIC profile. The WDIC profile for hole + crack is no more symmetric (Figure 5c) since the damaged rivet hole (hole + crack) is not symmetric about the line of incidence. In order to clearly identify the effect of damage in the plate, the scattered fields can be separated from the total fields. To find out the scattered field due to the presence of the hole, incident wave fields (wave fields due to pristine plate) needs to be subtracted from the total wave field (incident + scattered) and the corresponding WDIC profile of the hole (only) is plotted in Figure 6a. Similarly, to find out the scattered field due to the presence of the crack (only), the scattered wave field of the hole (only) and the incident wave fields needs to be subtracted from the total wave fields (due to hole with crack + incident). The corresponding WDIC profile of scattered S0 Lamb mode and SH wave for the crack (only) is plotted in Figure 6b. The WDIC profiles indicate that the magnitude of WDIC reaches the larger value at certain azimuth angles Φ.

### 6.3. Frequency Domain Variation of WDIC at Different Azimuthal Positions

**Figure 7:**
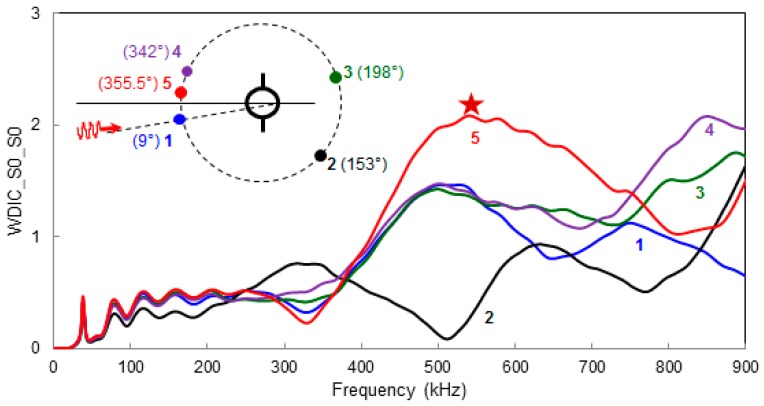
Frequency domain variation of WDIC_S0_S0_ at different azimuthal positions (θ=9°).

Frequency domain variation for a certain Lamb wave mode (*S*0 mode) incident from a particular direction (θ=9°) to the cracked rivet hole is shown in Figure 7. Five different azimuthal locations are picked arbitrarily to show the frequency domain variation of the WDICs. It can be noticed that at a certain location, a certain frequency of excitation provides the largest magnitude of WDIC. This frequency may be termed as sensitive frequency of that location. However, at a certain sensitive frequency, all the azimuthal locations are not necessarily equally sensitive. Thus the selection of frequency of excitation as well as the location is important in order to capture the damage signature. By comparing all azimuthal location, it is possible to select a certain frequency that corresponds to the highest magnitude of the WDIC, for example, in this particular case, the star marked frequency (f=538 kHz) can be the most sensitive excitation frequency at location 5 (“most sensitive location”).

### 6.4. Frequency Domain Variation of WDIC for Multiple Incident Directions

**Figure 8:**
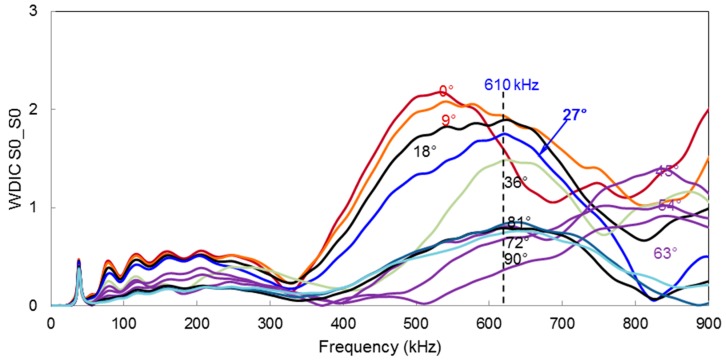
Frequency domain variation of WDIC_S0_S0_ for multiple incident directions.

The frequency domain variation of WDIC can be extended at the most sensitive locations for different directions of incident Lamb waves. Figure 8 illustrates all possible directions of incident Lamb waves for the multiple-rivet hole lap joint. This plot can be used to find out the optimum center frequency of excitation for a particular incident direction of Lamb waves.

For example, when *S*0 Lamb wave mode hit the cracked rivet hole at θ=27°, the excitation center frequency f=610 kHz corresponds the highest WDIC. In Section 7, we will show how the magnitude of WDIC affects the physical signal.

The similar frequency domain plots for scattered *A*0 mode can be produced from the scatter cube of *A*0 Lamb wave mode incident, but it is not shown here for the sake of brevity.

### 6.5. Azimuthal Variation of WDIC

**Figure 9:**
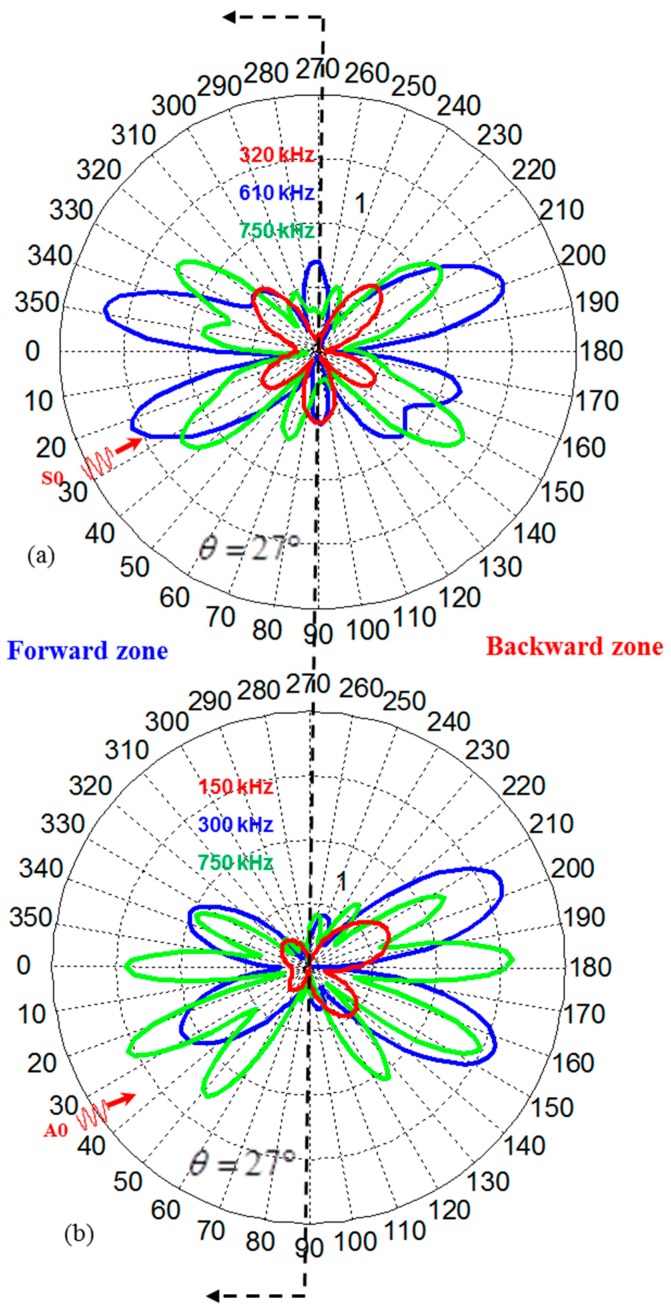
Azimuthal variation of (**a**) ${\text{WDIC}}_{S0\_S0}$; (**b**) ${\text{WDIC}}_{A0\_A0}$ at different frequencies.

The polar plot refers to the azimuthal variation of WDIC and can be used to identify the locations where WDIC reaches the maximum. The azimuthal variation of WDIC for symmetric and antisymmetric Lamb wave incident at 27° to the rivet hole cracks are shown in Figure 9. As the frequency changes, the WDIC profile for both symmetric and antisymmetric Lamb wave changes. It is possible to get multiple sensitive locations around the damage for a certain frequency of transmitting signal.

Sometimes, it may be important to classify the sensitive locations into two zones (forward and backward) because depending on applications there could be space limitations to install the sensors in a certain zone. For the problem of lap joint in a real life structure, it is convenient to install the PWASs in the forward zone only and, thus, the most sensitive locations in the forward zone will come into play for optimum design of the sensors.

## 7. Simulated Time Domain Signals Using Combined FEM-Analytical Model

**Figure 10:**
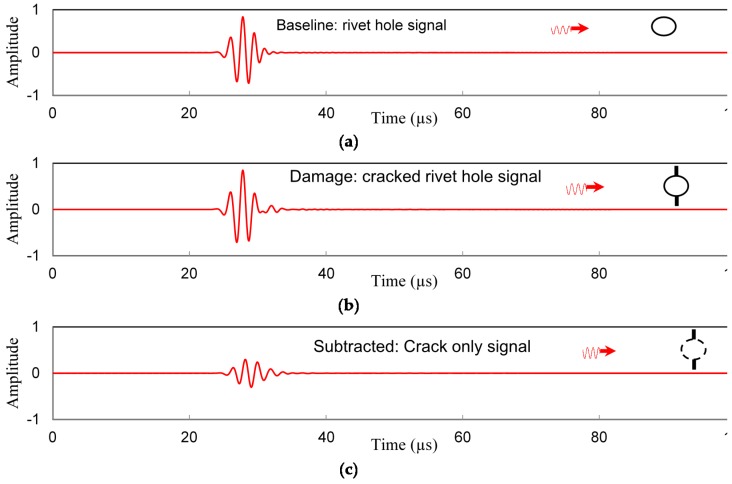
The wave fields for (**a**) Hole; (**b**) Hole + Crack (**c**) Crack only (f=538 kHz, θ=0°).

In this section we will show how the timedomain signals varies with WDIC and the selection of the frequency and locations of the sensors. Three count Hanning window modulated signal from a transmitter PWAS is used. The actuator is located at 100 mm away from the damage and the receiver is 30 mm away from the damage. The signal extraction process is illustrated in Figure 10. When there is a hole in the plate, the signal in the receiver PWAS behaves as shown in Figure 10a, and in presence of cracks in the rivet holes, the receiver signal changes as shown in Figure 10b. By subtracting the two signals, the receiver signals due to the cracks is be obtained as shown in Figure 10c. This illustration is shown for a particular frequency of 538 kHz and when Lamb waves incident horizontally to the rivet hole with cracks.

**Figure 11:**
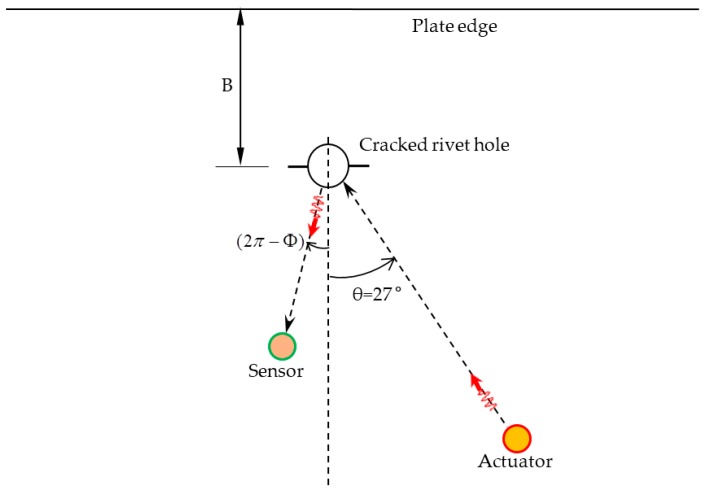
A simplified case of the multiple-rivet-hole problem.

In this present research, we consider a simplified case of the multiple-rivet-hole problem as shown in Figure 11 where the actuator is located at θ=27°. A single rivet hole is considered for determining the optimum location of the sensor and the optimum frequency of excitation. Furthermore, the rivet hole is considered to be located sufficiently far from the edge of the plate (the length, B, is large) so that: (1) the reflection from the edge dies out sufficiently before it reaches the sensor; and (2) the very low amplitude reflected signal would be seen in the trailing part of the main signal and can be easily discarded. When there are multiple rivet holes, there will be mutual interactions of the scattered waves among the rivet holes. When the plate edge is located close to the rivet holes, the plate boundary will act as a secondary source of the scattered waves and distance B will have an effect on the overall result. These complexities have not been included in this present study and will be focused on in our future research.

**Figure 12:**
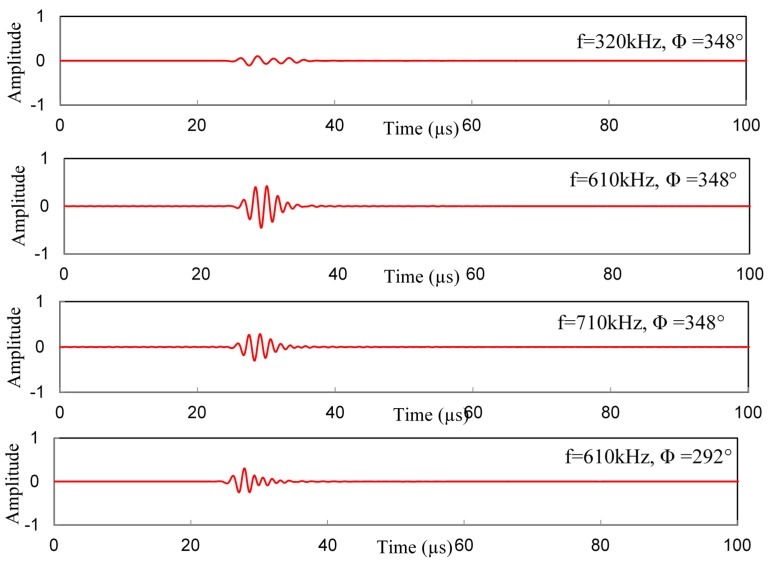
Sensing signals for different sets of frequency–location (θ=27°).

The effect of different frequency–location parameters on the receiver signal is shown in Figure 12. The four different sets of parameter are selected based on Figure 8 and Figure 9. Transmitting of the *S*0 Lamb mode from an actuator at θ=27° is used for this illustration. Figure 8 shows that at most sensitive location (Φ=348°), WDIC reaches the maximum at around 610 kHz frequency. At the same location, two other frequencies (320 kHz, 710 kHz) are chosen to show the differences in the receiver signals as illustrated in Figure 12. It shows that 610 kHz excitation frequency gives the strong sensing signal at that location (Φ=348°), hence it becomes the most sensitive frequency. Additionally, by selecting the most sensitive frequency (610 kHz), if we change the location of the receiver sensor where WDIC magnitude is smaller (Figure 9) we can end up with a weak signal. Thus, for a particular Lamb wave incident (θ=27°), the optimum center frequency of excitation (610 kHz) and location of the receiver sensor (Φ=348°) can be selected for better detection of the cracks in the rivet holes.

## 8. Conclusions

Exact analytical formulation has been used throughout the structure, except for the local damage area, which was analyzed using the finite element method. In order to analyze the multiple-rivet-hole lap joint cracks, the Lamb waves have been impinging on the damage from all possible directions. Both symmetric and antisymmetric fundamental Lamb wave modes (*S*0 and *A*0) were used for the analyses. SH waves appeared in the scattered waves besides the Lamb waves. Due to the non-axisymmetric nature of the problem, the wave damage interaction coefficient (WDIC) had non-axisymmetric behavior around the damage. The scatter cubes were produced for the scattered waves to accommodate the 3D interaction (frequency-incident direction-azimuthal direction) of Lamb waves with the rivet hole cracks. From the frequency domain analysis and the azimuthal variation of the WDIC, the proper locations of the sensors and center frequency of excitation have been obtained through the demonstration of a particular case (θ=27°). The physical time–domain signals were obtained using a piezoelectric transmitter for different sets of frequency–location. The optimum selection of the location of the sensor and the center frequency of excitation gave a strong signal that confirms better detection of the cracks in the rivet hole. The optimum parameters can be used for making an algorithm of NDE/SHM unit for inspecting the multiple-rivet-hole lap joint.

## 9. Future Work

An experiment may be designed based on the simulated results to detect the cracks in the rivet holes with Lamb wave incident from multiple directions. The design may be extended for making an algorithm for the multiple-rivet-hole lap joint and detecting the cracks in any of the rivet holes. The research may be further extended by considering the interactions among the rivet holes and the boundary reflections from the edges. In the bonded section of the plate, there may occur some wave leakages through the bonded rivet, thus, the thickness of the bonded plate will come into play. The wave leakages may be considered while obtaining the optimum parameters.
